# Impact of Poor Sleep Quality on the Academic Performance of Medical Students

**DOI:** 10.7759/cureus.4357

**Published:** 2019-04-01

**Authors:** Ganpat Maheshwari, Faizan Shaukat

**Affiliations:** 1 Internal Medicine, Jinnah Sindh Medical University, Karachi, PAK; 2 Internal Medicine, Jinnah Postgraduate Medical Center, Karachi, PAK

**Keywords:** pittsburgh sleep quality index, sleep quality, medical students, academic performance, grade point average, poor sleeper, daytime dysfunction

## Abstract

Introduction: Adequate sleep has a crucial role in enhancing cognitive skills especially memory retention. Poor night time sleep quality and the consequent daytime sleepiness affect physical and cognitive health of students and their academic performance. The aim of this study is to find whether or not poor academic performance is a consequence of poor sleep quality among Pakistani medical students.

Methods: It was an observational, cross-sectional study conducted with undergraduate medical students. Pittsburgh Sleep Quality Index (PSQI) was used to assess the sleep quality. Academic performance was assessed by mean grade point average (GPA) of the students. The data were analyzed using the Statistical Package for the Social Sciences (SPSS) 22.0 (IBM Corp., Armonk, NY, USA).

Results: There were 512 (64.24%) students with global PSQI score ≥5 indicating poor sleep quality. The mean GPA of poor sleepers was 2.92 ± 1.09 which was significantly lower than that of good sleepers (p < 0.0001). In the group of students who scored lower GPA (2.0-2.7), 28.2% had very bad subjective sleep quality, 29.05% had sleep latency of 16-30 min, 29.4% had sleep duration of <5-7 h, 27.8% had sleep efficiency of <85%, and 37.7% experienced daytime dysfunction almost every day.

Conclusion: Medical students of Pakistan have poor sleep quality which has a negative impact on their academic performance. Adequate sleep is essential to refresh the students every day and help them in learning and memory processing. Medical students and their facilitators should comprehend the negative effects of sleep deprivation on student academics and should take adequate measures to improve the sleep quality of students.

## Introduction

Adequate sleep of high quality and optimum duration facilitates memory processing and learning. It helps maintain concentration, executive cognitive functions, sensorimotor integration, and memory processing [1]. Sleep patterns and habits are different for different individuals depending on their age, occupational demands, social engagements, psychiatric and somatic conditions, and also individual physiological characteristics [2-3].

Young and old adults are recommended to sleep for seven to nine hours every night [4]. However, recent literature predominantly shows how most of the young adults are sleeping for less than the recommended duration [5-6]. The relationship of sleep inadequacy with stress is that of a chain reaction. Studies have established that sleep disturbances are, at times, caused by psychosocial stressors and also that psychosocial stressors culminate in sleep inadequacy [2-3, 5-6]. Disturbed and inadequate sleep leads to judgement impairment, agitation, irritability, and inability to process information in the short term, and in the long term, it can contribute to cardiometabolic disorders and even increased mortality [7].

One of the high-risk groups, established in the literature, for being affected by disorders of sleep are students [2-3, 5]. A high propensity has been seen in young students attending medical schools [6-7]. In medical students, various studies have been conducted to assess sleep disturbances and their results vary based on their years of education and their geodemographic locations. In Chinese medical students, approximately 90% or more reported in-class daytime sleepiness [8] and the percentage was 35.5% for Malaysian students which was more common in clinical students [9]. In Hong Kong, medical students had a mean night-time sleep duration of 6.6 ± 1.2 h; 70% of them complained of sleep deprivation [10]. Poor sleep quality has been reported in 16% of Malaysian medical students [9], 40.6% of Iranian medical students-with highest prevalence in their interns [11], 62.6% of Indian students [12], and 77% of Pakistani medical students [13].

Literature has extensively reported high prevalence of poor sleep quality among medical students. However, not many researchers have studied the consequences of inadequate sleep quality. It has been proposed that sleep deprivation causes depression, agitation, apathy, and poor academic performance in students [14]. Reduced total sleeping hours have been associated with declining academic performance [15].

As stated earlier, studies have reported the frequency of poor sleepers among Pakistani medical students and its association with academic stress [13]; studies have not yet reported the impact of proper sleep on the academic performance of these students. The aim of this study is to find any correlation between poor sleepers and their grade point average (GPA).

## Materials and methods

It was an observational, cross-sectional study conducted with undergraduate students in public medical university of Karachi, Pakistan. The study duration was from 1st January till 15th March 2019. Students of both genders, and all five years of education were included. With approximately 350 students in each batch, all 1750 MBBS students were invited, out of which 980 students responded. There were 183 incomplete responses which were excluded. Remaining 797 responses were included in the study (response rate: 45.54%).

A structured questionnaire, consisting of two sections, was distributed. The first section comprised demographic information including gender, age, and GPA of every semester. The second part comprised Pittsburgh Sleep Quality Index (PSQI). PSQI is an efficient measure of the quality and pattern of sleep. It assesses sleep quality on seven components-subjective sleep quality, sleep latency, sleep duration, habitual sleep efficiency, sleep disturbances, use of sleeping medication, and daytime dysfunction. Its scores range from minimum zero to maximum 21. The combined score of all seven components is termed as “global score of PSQI.” Global PSQI score ≥5 signifies “poor sleep quality.” The PSQI has internal consistency and a reliability coefficient (Cronbach's alpha) of 0.83 for its seven components [16].

The SPSS for Windows, version 22.0 (IBM Corp., Armonk, NY, USA) was used to enter and analyze the data. Demographic data were categorized to calculate frequencies and percentages. All components of PSQI were categorized as mentioned by Smyth [16]. Mean GPA was calculated for every student. GPA was recorded as a numerical variable. It was then transformed to categorical variable by sub dividing into three groups (< 2.7, 2.71-3.40, and > 3.41). All scores of PSQI were compared against GPA. Chi square was calculated for correlation between PSQI score and GPA. A p value of ≤0.05 was taken as significant.

## Results

Out of the 791 students, 531 (66.62%) were females and 266 (33.37%) were males. Mean GPA was 3.08 ± 1.12 for the entire study sample. There were 241 (30.23%) students with GPA less than 2.71, 312 (39.14%) students with GPA 2.71-3.40 and 244 (30.61%) with GPA 3.41-4.00.

Poor sleep quality was witnessed in 512 (64.24%) students. Their mean GPA was 2.92 ± 1.09. There were 285 (35.76%) students who were not poor sleepers and their PSQI score was <5. Their mean GPA was 3.31 ± 1.49. Poor sleepers had significantly lower mean GPA (p < 0.0001).

All seven components of PSQI were compared with the average GPA of the students. Most of the students with lower GPA (2.0-2.7) had very bad subjective sleep quality (28.2%), sleep latency of 16-30 min (29.05%), sleep duration of <5-7 h (29.4%), and sleep efficiency of 75%-84% (27.8%). Most of these students experienced sleep disturbances once or twice a week (29.8%), had not used sleep medication in the past one month (87.14%), but experienced daytime dysfunction almost every day (37.7%). The comparison of their sleep quality parameters to groups with higher GPAs is shown in Table 1.

**Table 1:** Sleep quality as assessed by PSQI in medical students correlated with their mean GPA (n = 791). PSQI, Pittsburgh Sleep Quality Index; GPA, grade point average.

Components of PSQI	GPA 2.0 to 2.7 (n = 241)	GPA 2.71 to 3.40 (n = 312)	GPA 3.41 to 4.00 (n = 244)
Subjective sleep quality			
Very good	52 (21.58%)	93 (27.56%)	61 (33.20)
Fairly good	74 (19.50%)	86 (19.87%)	81 (33.20%)
Fairly bad	47 (19.50%)	62 (19.87%)	57 (23.36%)
Very bad	68 (28.22%)	71 (22.76%)	45 (18.44%)
Sleep latency			
≤ 15 min	56 (23.24%)	71 (22.76%)	71 (29.10%)
16-30 min	70 (29.05%)	76 (24.36%)	65 (26.64%)
31-60 min	59 (24.48%)	78 (25.00%)	57 (23.36%)
>60 min	56 (23.24%)	87 (27.88%)	51 (20.90%)
Sleep duration			
>7 h	31 (12.86%)	42 (13.46%)	36 (14.75%)
6-7 h	71 (29.46%)	86 (27.56%)	90 (36.89%)
5-6 h	68 (28.22%)	107 (34.29%)	67 (27.46%)
<5 h	71 (29.46%)	77 (24.68%)	51 (20.90%)
Habitual sleep efficiency			
>85%	34 (14.11%)	53 (16.99%)	39 (15.98%)
75%-84%	67 (27.80%)	102 (32.69%)	81 (33.20%)
65%-74%	61 (25.31%)	74 (23.72%)	74 (30.33%)
<65%	79 (32.78%)	83 (26.60%)	50 (20.49%)
Sleep disturbances			
Not during the past month	50 (20.75%)	91 (29.17%)	59 (24.18%)
Less than once a week	49 (20.33%)	88 (28.81%)	83 (34.01%)
Once or twice a week	72 (29.88%)	60 (19.23%)	59 (24.18%)
Three or more times a week	70 (29.05%)	73 (23.40%)	43 (17.62%)
Use of sleep medication			
Not during the past month	210 (87.14%)	271 (86.86%)	231 (94.67%)
Less than once a week	19 (7.88%)	28 (8.97%)	11 (4.51%)
Once or twice a week	10 (4.15%)	12 (3.85%)	2 (0.82%)
Three or more times a week	2 (0.83%)	1 (0.32%)	0 (0.00%)
Daytime dysfunction			
1-2 days	53 (21.99%)	81 (25.96%)	72 (29.51%)
3-4 days	50 (20.75%)	76 (24.36%)	71 (29.10%)
5-6 days	47 (19.50%)	73 (23.40%)	58 (23.77%)
Everyday	91 (37.76%)	82 (26.28%)	43 (17.62%)

## Discussion

Adequate and efficient sleep plays a crucial role in learning and memory. It is important for students to sleep well in order to perform well in academics. The results of this study are very alarming. Most students with lower average GPA reported very bad subjective sleep quality, had a sleep latency of 16-30 min, sleep duration of <5-7 h, sleep efficiency of 75%-84%, and experienced daytime dysfunction almost every day.

The study provides robust evidence regarding the association of sleep disturbances with declining pattern in mean GPA scores. However, the study has its limitations too. It has not taken into account or excluded other factors such as exam stress, exam difficulty, etc. that can contribute to poor academic performance. Also, it was conducted among students of only one medical college and cannot be generalized for the entire population. This is a cross-sectional study which only shows an association and, in no manner, has determined causality.

Even with our best efforts, we could not find any local data published. However, there is substantial evidence from other countries regarding the delirious effects of sleep deprivation on students’ academic performance. Among Saudi medical students, sleep disturbances including insomnia, frequent awakenings, and falling asleep after 30 min of going to bed were common [6]. Similarly, Indian students who slept for shorter duration reported lower GPAs and poor memory and concentration [17].

Philips in 2017 in his research stated that for every 10-point increase in sleep regularity index, a 0.10 increase in GPA has been observed. Furthermore, negative linear correlations have been observed between GPA and sleep onset time, wake time, and mid-sleep time [18]. In Sudanese medical students, there were significant differences in the overall sleep quality, subjective sleep rating, bedtime later than midnight, sleep latency, and daytime dysfunction between students who scored an “A” in the exams and those who scored a “C.” Their “A” scoring group had a higher mean sleep duration than the “C” scoring group [19]. In Iran, 85% of the medical students who scored a GPA of 2.99 or less were poor sleepers on PSQI [20]. If looked together, the results suggest a strong negative relationship between sleep quality and academic performance of the medical students.

## Conclusions

Medical students are continuously under high academic stress and pressure. Adequate sleep is essential to refresh them every day and help in learning and memory processing. Sleep disturbances are common in medical students and worsen their academic performance. Medical students and their facilitators should comprehend the negative effects of sleep deprivation on student academics and should take adequate measures to improve the sleep quality of students.
